# Inborn errors of immunity in Low German Mennonite communities in Mexico: a case series and narrative literature review

**DOI:** 10.3389/fimmu.2026.1862004

**Published:** 2026-06-24

**Authors:** Luisa Berenise Gámez-González, Saúl Oswaldo Lugo-Reyes, Rogelio Guzmán-Cotaya, Sara Elva Espinosa-Padilla, Luis Enrique Murguía-Favela, Marco Antonio Yamazaki-Nakashimada

**Affiliations:** 1 Immunology Department, Specialties Children's Hospital of Chihuahua, Chihuahua, Mexico; 2 Faculty of Medicine and Biomedical Sciences, Autonomous University of Chihuahua, Chihuahua, Mexico; 3 Immune Deficiency Laboratory, National Institute of Pediatrics, Health Secretariat, Mexico City, Mexico; 4 Independent Practitioner, Mérida, Yucatán, Mexico; 5 Section of Pediatric Hematology and Immunology, Alberta Children’s Hospital, University of Calgary, Calgary, AB, Canada; 6 Clinical Immunology Department, National Institute of Pediatrics, Mexico City, Mexico

**Keywords:** endogamy, founder effect, inborn errors of immunity, Low German Mennonite, Mennonite, newborn screening, severe combined immunodeficiency

## Abstract

**Background:**

Inborn errors of immunity (IEI) comprise a genetically heterogeneous group of disorders with increased prevalence in consanguineous and endogamous populations due to founder effects. Low German Mennonite (LGM) communities in Mexico represent a high-risk population characterized by genetic isolation and an increased frequency of autosomal recessive conditions. Although IEI have been reported in LGM populations from Canada and Europe, systematic data from Mexican LGM communities remain limited. This study describes four novel clinical cases of IEI in LGM patients evaluated at two tertiary referral centers in Mexico and contextualizes these findings within a comprehensive review of previously reported IEI in this population.

**Methods:**

A retrospective case series was conducted at two tertiary pediatric referral centers in Mexico between 2020 and 2025, including four patients from LGM communities with clinically and/or genetically confirmed IEI. Clinical, immunological, and genetic data were extracted from medical records. Genetic diagnoses, when available, were established using next-generation sequencing or whole-exome sequencing, with variant classification according to ACMG criteria. A narrative literature search was performed in PubMed, Embase, SciELO, and Web of Science to identify previously reported IEI cases in LGM populations.

**Results:**

Four patients with distinct IEI were identified: one with glucose-6-phosphatase catalytic subunit 3 (G6PC3) deficiency, one with X-linked agammaglobulinemia (XLA) due to a pathogenic variant in the Bruton tyrosine kinase (BTK) gene, and two with severe combined immunodeficiency (SCID). All patients required disease-specific therapy, including granulocyte colony-stimulating factor, immunoglobulin replacement, and/or hematopoietic cell transplantation. The literature review identified a broad spectrum of IEI in LGM populations, including cellular and humoral immunodeficiencies, phagocyte disorders, DNA repair defects, autoinflammatory conditions, and bone marrow failure syndromes.

**Conclusions:**

This exploratory case series contributes to the growing body of evidence on IEI in LGM populations and highlights the potential impact of founder variants in this community. Given the small sample size and retrospective design, generalization of these findings requires prospective, population-based validation. Early diagnosis through newborn screening, accessible genetic testing, and timely clinical intervention remains essential to improve outcomes.

## 1 Introduction

Inborn Errors of Immunity (IEI) comprise a heterogeneous group of genetic disorders characterized by dysfunction in one or more components of the immune system, predisposing individuals to infections, autoimmunity, autoinflammation, malignancy, and allergies (1).

IEI occur more frequently in endogamous and consanguineous populations, where founder effects and genetic isolation contribute to an increased prevalence of rare inherited conditions.

The Low German Mennonite (LGM) community is a population group that originated in Central Europe more than 500 years ago, with migrations spreading to various countries, including North America in the early 18th century (2).

The Low German Mennonite (LGM) community in Mexico is concentrated primarily in the northern states of Chihuahua, Durango, and Tamaulipas, with approximately 65 established settlements (3, 4). These communities originate from approximately 1,300 Old Colony Mennonite families who emigrated from Manitoba, Canada, to Mexico between 1922 and 1926 in search of greater religious and cultural autonomy. The LGM population is characterized by strict endogamy, limited integration with the broader Mexican society, geographic clustering in rural agricultural colonies, and the use of Low German (Plautdietsch) as the primary spoken language — factors that together contribute to reproductive isolation and a restricted gene pool. This demographic structure creates ideal conditions for the propagation of founder alleles across generations and the accumulation of rare autosomal recessive conditions, including inborn errors of immunity.

Several studies have identified pathogenic genetic variants in this population that contribute to the development of rare immunological diseases. Among these conditions, SCID is associated with pathogenic variants in genes encoding for either adenosine deaminase (*ADA*) or the CD3δ chain of the T cell receptor (*CD3D*) (5–9). Other IEI described in this population include T-cell signaling defects such as *ZAP70* deficiency (10–15), as well as DNA repair disorders including ataxia-telangiectasia (*ATM*) (16–20) and Bloom syndrome (*BLM*) (21, 22). In addition, *G6PC3* deficiency, a cause of severe congenital neutropenia, has also been described (23).

However, despite progress in identifying these disorders, a clear epidemiological understanding of the prevalence of IEI in LGM communities in Mexico remains lacking. This study aims to describe four clinical cases of IEI in LGM patients from Mexico and to contextualize these findings within a comprehensive narrative review of the literature. By integrating clinical, immunological, and genetic data, this work seeks to highlight the importance of early recognition of IEI in genetically isolated populations and to contribute to a better understanding of their underlying genetic architecture.

## 2 Materials and methods

### 2.1 Study design and population

This retrospective case series analyzed four representative cases of IEI in LGM communities in Mexico. Patients were identified from medical records at the Specialties Children’s Hospital of Chihuahua, Mexico, and the National Institute of Pediatrics in Mexico City, Mexico, covering the period from 2020 to 2025. All patients met the diagnostic criteria for IEI according to the International Union of Immunological Societies (IUIS) 2024 classification (1). Genetic testing was performed when feasible. Given the retrospective design and limited sample size, this study is descriptive and exploratory in nature; it is not intended to provide population-level prevalence estimates or to formally establish new genotype–phenotype correlations.

### 2.2 Data collection

Clinical, laboratory, and genetic data were retrieved from medical records, including demographic information, family history, immunological workup, genetic testing results, and treatment outcomes. When available, additional details were obtained through structured interviews with family members.

### 2.3 Genetic analysis

Genetic testing was conducted using a next-generation sequencing panel (Invitae/LabCorp) targeting IEI-related genes or by whole-exome sequencing (WES). Identified genetic variants were classified based on the pathogenicity criteria established by the American College of Medical Genetics and Genomics (ACMG) (24).

### 2.4 Narrative literature search

A narrative literature search was conducted to identify previously reported cases of inborn errors of immunity (IEI) in Low German Mennonite (LGM) populations. Searches were performed in PubMed/MEDLINE, Embase, SciELO, and Web of Science, supplemented by hand-screening of the Amish, Mennonite, and Hutterite Genetic Disorders Database (Biochemical Genetics Laboratory, London, Ontario) (25). The search covered all records up to August 2025, with no lower date limit. Combinations of the following terms were used in titles, abstracts, and keywords: (‘inborn errors of immunity’ OR ‘primary immunodeficiency’ OR ‘immunodeficiency’ OR ‘genetic disorders’) AND (‘Mennonite’ OR ‘Low German Mennonite’ OR ‘Amish’ OR ‘Hutterite’). Inclusion criteria were: (i) original studies (case reports, case series, cohort studies, and registry analyses) reporting clinical, immunological, and/or genetic data on IEI in LGM individuals; (ii) publications in English or Spanish; (iii) human studies of any age. Exclusion criteria were: (i) reports without identifiable Mennonite ancestry; (ii) duplicate publications or overlapping patient series (the most complete report was retained); (iii) conference abstracts without sufficient clinical detail; and (iv) studies focused exclusively on non-immune genetic disorders. Approximately 120 records were initially identified across all databases and the supplementary source. After removal of duplicates and screening for relevance at the title/abstract and full-text levels, 36 articles were included for qualitative narrative synthesis. Records were independently screened by two authors (L.B.G. and M.A.Y.) at the title/abstract and full-text levels; disagreements were resolved by consensus with a third author (S.O.L.). Reference lists of all eligible full-text articles and relevant reviews were manually screened to identify additional studies. Given that the underlying literature consists predominantly of case reports and small case series with heterogeneous reporting standards, the synthesis was narrative rather than systematic, and quantitative meta-analysis was not attempted.

### 2.5 Ethical considerations

This study was conducted in accordance with the Declaration of Helsinki and was approved by the Institutional Review Board (IRB) of the Specialties Children’s Hospital of Chihuahua, México (protocol code CIMAD 0016), and was exempt at the National Institute of Pediatrics, Mexico City. Informed consent was obtained from patients or their legal guardians prior to data collection and genetic testing.

## 3 Results

### 3.1 Case 1

A male infant from the Old Colony Mennonite (LGM) community in Cuauhtémoc, Chihuahua, Mexico, was admitted at 2 months of age with severe pneumonia. One month later, he developed recurrent suppurative otitis media, oral ulcers, and persistent fever. He was treated with myringotomy and antimicrobial therapy; however, intermittent otorrhea continued.

At 15 months of age, he was referred to the Immunology Department for further evaluation. Severe neutropenia was documented. The hematological profile showed white blood cells (WBC) 6,800 cells/μL, neutrophils 80 cells/μL, lymphocytes 5,300 cells/μL, platelets 420,000 cells/μL, hemoglobin (Hb) 12.1 g/dL, and hematocrit (Hct) 36%. Immunoglobulin levels were within age-adjusted reference ranges (IgA 47 mg/dL, IgG 1,069 mg/dL, IgM 105 mg/dL, and IgE 21 IU/mL). Lymphocyte subpopulation analysis revealed CD3^+^ 6,722 cells/μL, CD4^+^ 3,394 cells/μL, CD8^+^ 2,466 cells/μL, CD16^+^/56^+^ 168 cells/μL, and CD19^+^ 591 cells/μL.

**Figure 1:**
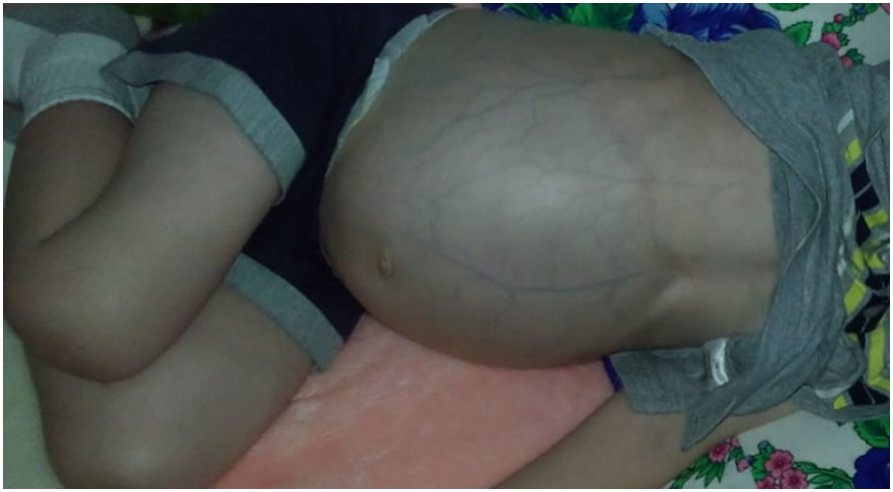
Abdominal collateral veins. Typical clinical manifestation in G6PC3 deficiency.

Bone marrow aspiration showed normocellular preserved hematopoietic niches, and no abnormal morphology. The M/E ratio was 1:3. Findings were inconclusive for congenital neutropenia. Exome sequencing revealed a homozygous pathogenic variant in the *G6PC3* gene (c.482G>A; p.Arg161Gln), confirming G6PC3 deficiency. Additional findings included atrial septal defect, cryptorchidism, and abdominal collateral veins (**Figure 1**). The patient was initiated on granulocyte colony-stimulating factor (G-CSF) therapy and remains clinically stable at 8 years of age.

### 3.2 Case 2

A 5-month-old male patient from a LGM community in Casas Grandes, Chihuahua, Mexico, presented with respiratory difficulty, oral candidiasis, and fungemia due to Candida parapsilosis. Immunological evaluation revealed a T^-^B^+^NK^+^ SCID immunophenotype (CD3^+^ 35 cells/μL; CD4^+^ 5 cells/μL; CD8^+^ 5 cells/μL; CD19^+^ 4,196 cells/μL; CD16^+^/56^+^ 737 cells/μL). Genetic testing was not performed. Based on the immunophenotype, clinical course, and the known high prevalence of CD3δ deficiency in Mexican LGM communities, a presumptive (molecularly unconfirmed) diagnosis of CD3D-associated SCID was established. The patient underwent HCT and is currently alive and clinically stable five years post-transplant.

### 3.3 Case 3

A 5-month-old male from a LGM community in Cuauhtémoc, Chihuahua, Mexico, presented with respiratory distress, myocarditis, and multisystem inflammatory syndrome associated with SARS-CoV-2 infection. He required broad-spectrum antibiotics and intravenous immunoglobulin (IVIG). He had a remarkable family history of a sibling who died at 5 months of age with a diagnosis of severe sepsis, and a female cousin who was previously diagnosed with CD3 delta deficiency, consistent with SCID.

Immunological evaluation revealed severe hypogammaglobulinemia (IgG 120 mg/dL, IgA <10 mg/dL, IgE 0 IU/mL, and IgM 27 mg/dL) and marked T-cell lymphopenia (CD3^+^ 40 cells/μL; CD4^+^ <20 cells/μL; CD8^+^ 44 cells/μL), with preserved B and NK cell counts (CD19^+^ 1,676 cells/μL; CD16^+^/56^+^ 1,096 cells/μL). The total lymphocyte count was 2,658 cells/μL, consistent with a T^-^B^+^NK^+^ SCID phenotype.

Genetic analysis using a targeted next-generation sequencing panel for primary immunodeficiencies (Invitae/LabCorp) identified a homozygous pathogenic variant in *CD3D* (c.202C>T; p.Arg68*). A heterozygous variant in *ADA* (c.424C>T; p.Arg142*) was also detected. Following an HCT from his 10/10 HLA-matched father at the age of 18 months, 100% donor chimerism of all lineages was achieved, with no acute or chronic graft-versus-host disease. Four years post-transplant, he remains clinically stable with full immune reconstitution, although global developmental delay persists.

### 3.4 Case 4

**Figure 2:**
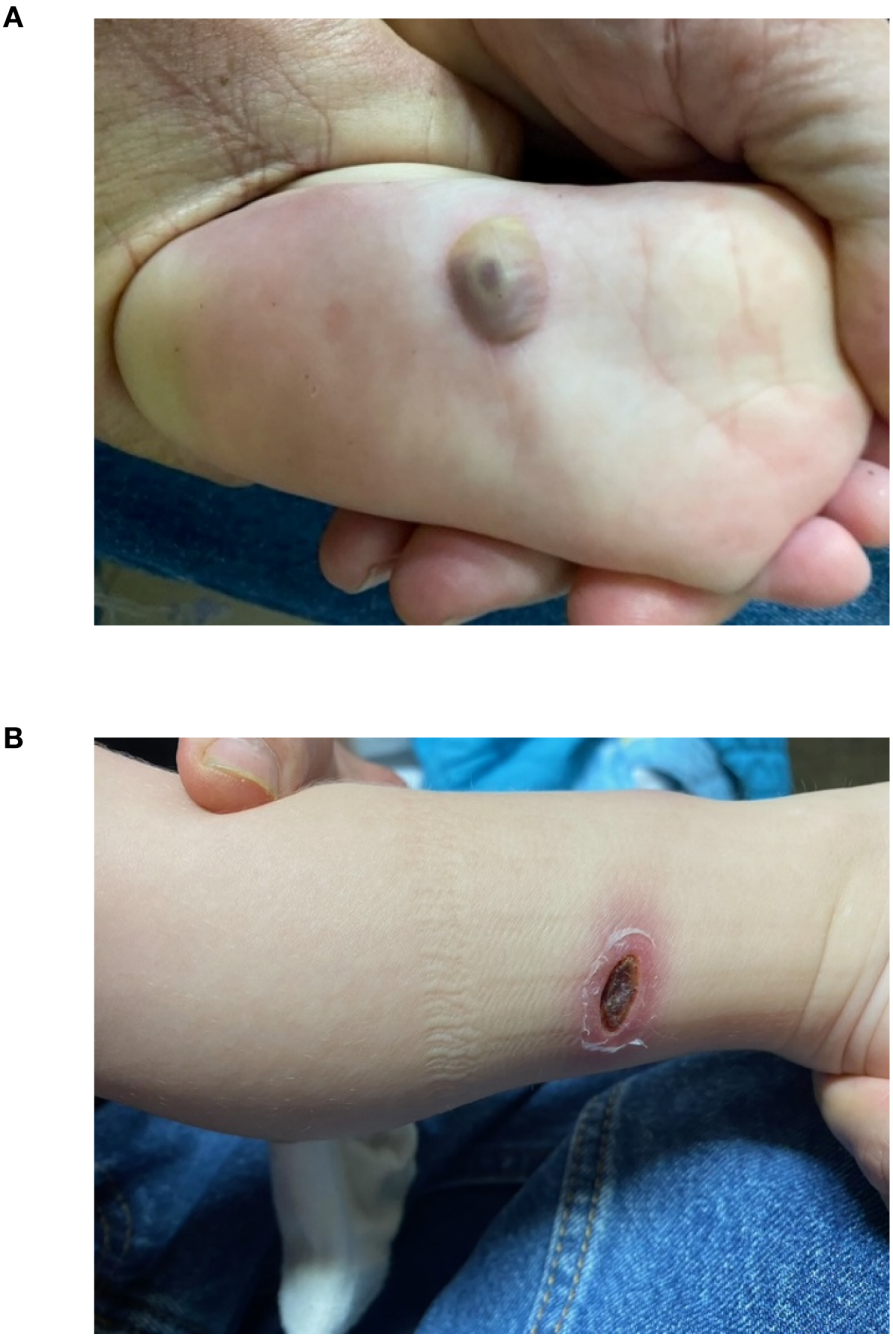
**(A)** Cutaneous abscess on the plantar surface of the foot. **(B)** Ulcer on the left lower extremity.

A 2-year-old male from an LGM family in northern Belize presented with chronic diarrhea, recurrent fevers, skin abscesses, and conjunctivitis. He was initially hospitalized with pneumonia, diarrhea, perianal ulcers, and a hydrocele (**Figures 2A, B**). Laboratory evaluation revealed anemia and thrombocytosis, with markedly reduced immunoglobulin levels (IgG <106 mg/dL, IgA <5 mg/dL, IgM 8.2 mg/dL). Flow cytometry demonstrated preserved T-cell counts with absent B cells. Whole-exome sequencing identified a hemizygous pathogenic variant in *BTK* (c.862C>T; p.Arg288Trp), confirming the diagnosis of X-linked agammaglobulinemia (XLA). Intravenous immunoglobulin therapy was initiated, and the patient remains clinically stable on regular intravenous immunoglobulin replacement at the most recent follow-up summary of the clinical, immunological, genetic, and follow-up characteristics of the four reported patients is presented in **Table 1**.

**Table 1:** Clinical, immunological, genetic, and follow-up summary of the four reported cases.

Feature	Case 1 — G6PC3 deficiency	Case 2 — clinically diagnosed CD3D-associated SCID	Case 3 — CD3D-associated SCID (molecularly confirmed)	Case 4 — X-linked agammaglobulinemia (XLA)
Sex/age at onset	Male/2 months	Male/5 months	Male/5 months	Male/2 years
Geographic origin (LGM)	Cuauhtémoc, Chihuahua, Mexico	Casas Grandes, Chihuahua, Mexico	Cuauhtémoc, Chihuahua, Mexico	Northern Belize
Presenting clinical features	Severe pneumonia, recurrent otitis media, oral ulcers	Respiratory failure, multifocal pneumonia, oral candidiasis, Candida parapsilosis fungemia	Respiratory distress, myocarditis, multisystem inflammatory features post SARS-CoV-2	Chronic diarrhea, recurrent fevers, skin abscesses, perianal ulcers, pneumonia
Relevant family history	None reported	Not reported	Sibling deceased at 5 months (severe sepsis); first cousin with molecularly confirmed CD3D deficiency	Not reported
Immunoglobulin profile	Normal for age (IgA 47, IgG 1069, IgM 105 mg/dL; IgE 21 IU/mL)	Not detailed; consistent with SCID	Hypogammaglobulinemia (IgG 120, IgA <10, IgM 27 mg/dL; IgE 0 IU/mL)	Profound hypogammaglobulinemia (IgG <106, IgA <5, IgM 8.2 mg/dL)
Lymphocyte subsets (cells/μL)	CD3 ^+^ 6722, CD4 ^+^ 3394, CD8 ^+^ 2466, CD19 ^+^ 591, CD16^+^/56 ^+^ 168; neutrophils 80	CD3 ^+^ 35, CD4 ^+^ 5, CD8 ^+^ 5, CD19 ^+^ 4196, CD16^+^/56 ^+^ 737 (T^-^B^+^NK^+^)	CD3 ^+^ 40, CD4^+^ <20, CD8 ^+^ 44, CD19 ^+^ 1676, CD16^+^/56 ^+^ 1096 (T^-^B^+^NK^+^)	Preserved T cells; absent B cells
Gene/variant	G6PC3 c.482G>A (p.Arg161Gln), homozygous	Presumed CD3D (no molecular testing)	CD3D c.202C>T (p.Arg68*), homozygous; incidental ADA c.424C>T (p.Arg142*) heterozygous	BTK c.862C>T (p.Arg288Trp), hemizygous
Diagnostic method	Whole-exome sequencing	Immunophenotype + clinical course (PIDTC 2022 criteria)	Targeted NGS panel (Invitae/LabCorp)	Whole-exome sequencing
ACMG classification	Pathogenic	Not applicable (clinical diagnosis)	Pathogenic	Pathogenic
gnomAD allele frequency	Extremely rare (enriched in LGM; Zhen et al., 2025)	Not applicable (clinical diagnosis)	Absent/extremely rare (absent in gnomAD v4)	Rare (low frequency; ClinVar/BTKbase)
ClinVar classification	Pathogenic	Not applicable	Pathogenic	Pathogenic
In silico predicted effect	Damaging (missense; p.Arg161Gln)	Not applicable	Nonsense/truncating (p.Arg68*)	Damaging (missense; p.Arg288Trp)
Treatment	G-CSF (ongoing)	Hematopoietic cell transplantation (HCT)	10/10 HLA-matched paternal HCT at 18 months; no GvHD	Regular intravenous immunoglobulin replacement
Follow-up duration	8 years	5 years post-HCT	4 years post-HCT	Ongoing replacement therapy
Current clinical/immunological status	Clinically stable on continuous G-CSF	Alive and clinically stable five years post-HCT	100% donor chimerism; full immune reconstitution; persistent global developmental delay	Alive and clinically stable on regular immunoglobulin replacement

ACMG, American College of Medical Genetics and Genomics; ADA, adenosine deaminase; BTK, Bruton tyrosine kinase; CD, cluster of differentiation; G-CSF, granulocyte colony-stimulating factor; gnomAD, Genome Aggregation Database; GvHD, graft-versus-host disease; HCT, hematopoietic cell transplantation; Ig, immunoglobulin (IgA, IgG, IgM, IgE); LGM, Low German Mennonite; MIS-C, multisystem inflammatory syndrome in children; NGS, next-generation sequencing; NK, natural killer cell; PIDTC, Primary Immune Deficiency Treatment Consortium; SARS-CoV-2, severe acute respiratory syndrome coronavirus 2; SCID, severe combined immunodeficiency; XLA, X-linked agammaglobulinemia.

## 4 Discussion

Genetically isolated and highly endogamous populations, such as the LGM community, are at substantially elevated risk for IEI due to the accumulation and propagation of founder pathogenic variants (26). The present case series illustrates this risk through four distinct IEI identified in LGM patients from Mexico, caused by variants in *CD3D*, *BTK*, and *G6PC3*, and contributes to the growing characterization of IEI in this population. Previously reported inborn errors of immunity in Low German Mennonite populations are summarized in **Table 2**.

**Table 2:** Reported inborn errors of immunity in Low German Mennonite populations.

Disorder	Gene	Inheritance	Founder variant	Predominant phenotype	Country/setting	Key references
ADA-SCID	ADA	AR	Splicing/nonsense R142X (exon 5 skipping)	T^-^B^-^NK^-^ SCID; severe opportunistic infections; responsive to ERT and HCT	Canadian LGM	(9)
CD3D-SCID	CD3D	AR	c.202C>T (p.Arg68*)	T^-^B^+^NK^+^ SCID; early respiratory/opportunistic infections; HLH and attenuated MIS-C reported	Mexican LGM (predominant); Canadian LGM	(5–8, 30)
Hypomorphic RAG1 SCID	RAG1	AR	c.527G>T (homozygous)	Late-onset CID with CMV viremia, cryptosporidiosis, and IBD	LGM (North America)	(33)
ZAP-70 deficiency	ZAP70	AR	c.1624-11G>A (splice site); compound heterozygosity described	Selective CD8^+^ T-cell deficiency; clinically severe immunodeficiency; curative HCT	Canadian, US, and Mexican LGM	(10–15)
X-linked agammaglobulinemia (XLA)	BTK	XLR	c.862C>T (p.Arg288Trp), hemizygous	Profound hypogammaglobulinemia; absent B cells; sinopulmonary and GI manifestations	LGM (Belize; first report — present series)	(35); present series
G6PC3 deficiency	G6PC3	AR	c.482G>A (p.Arg161Gln)	Severe congenital neutropenia; superficial venous pattern; congenital heart defects; urogenital anomalies	Argentinian and Mexican LGM	(23, 36); present series
p14/LAMTOR2 deficiency	LAMTOR2	AR	Homozygous 3’ UTR variant	Short stature, hypopigmentation, recurrent infections, severe neutropenia responsive to G-CSF	Mennonite family (Germany)	(37)
A-T Winnipeg (atypical ATM)	ATM	AR	c.6200C>A (p.Ala2067Asp)	Early-onset dystonia, late ataxia, no telangiectasias; high cancer risk; radiosensitivity	Canadian LGM	(16–20)
Bloom syndrome	BLM	AR	Biallelic BLM variants	Chromosomal instability, growth failure, cancer predisposition	Mennonite siblings	(21, 22)
Mevalonate kinase deficiency	MVK	AR	c.803T>C (p.Ile268Thr)	Autoinflammatory spectrum: MKD to neonatal mevalonic aciduria with cholestasis	Canadian LGM	(38, 39)
Fanconi anemia (FANCC)	FANCC	AR	c.67delG (Dutch ancestral founder)	Mild skeletal phenotype, bone marrow failure, cancer predisposition	Dutch, Canadian Manitoba, and Mexican (Tamaulipas) LGM	(40, 41)
Telomere biology disorder (TERT)	TERT	AD	K570N (p.Lys570Asn), heterozygous; haploinsufficiency	Dyskeratosis congenita spectrum; pulmonary fibrosis; bone marrow failure	LGM (reported)	(42, 43)

AD, autosomal dominant; AR, autosomal recessive; A-T, ataxia-telangiectasia; CMV, cytomegalovirus; ERT, enzyme replacement therapy; G-CSF, granulocyte colony-stimulating factor; GI, gastrointestinal; HCT, hematopoietic cell transplantation; HLH, hemophagocytic lymphohistiocytosis; IBD, inflammatory bowel disease; LGM, Low German Mennonite; MIS-C, multisystem inflammatory syndrome in children; MKD, mevalonate kinase deficiency; NK, natural killer cell; SCID, severe combined immunodeficiency; UTR, untranslated region; XLR, X-linked recessive.

The historical migration of approximately 1,300 LGM families from Manitoba, Canada, to northern Mexico established a genetically bottlenecked population carrying discrete founder alleles (3, 4), accounting for the recurrent identification of identical pathogenic variants across unrelated LGM families in Canada, the United States, and Mexico. A founder variant — defined as a pathogenic variant at disproportionately elevated frequency due to common ancestry — enables targeted genotyping as a cost-effective diagnostic strategy, a principle confirmed across multiple IEI categories spanning T- and B-cell deficiencies, phagocyte disorders, DNA repair defects, autoinflammatory conditions, and bone marrow failure syndromes in LGM communities (26–28).

### 4.1 Severe combined immunodeficiency: ADA and CD3D founder variants (cases 2 and 3)

ADA-SCID was the first LGM founder IEI characterized variant. Santisteban et al. identified the splicing defect R142X (exon 5 skipping) in two unrelated Canadian LGM families, establishing the founder effect for this condition (9). The benefit of TREC-based NBS was subsequently demonstrated when an LGM neonate was identified at nine days of life with a markedly reduced TREC value (0.35 copies/μL), enabling pre-symptomatic enzyme replacement therapy before definitive HCT (29).

The CD3D c.202C>T (p.Arg68*) founder variant has emerged as the predominant cause of SCID in Mexican LGM communities (case 3), with multiple reports documenting T^−^B^+^NK^+^ SCID presenting in the first months of life with severe opportunistic infections including *Pneumocystis jirovecii* pneumonia, HLH, and high transplant-related mortality (5, 7, 30). Marcus et al. analyzed outcomes in 13 patients with CD3δ deficiency who underwent HCT across seven international centers — eight of whom were LGM children — identifying HLA match, conditioning regimen, and early diagnosis as key prognostic factors (6).

A clinically diagnosed/probable CD3D-associated SCID in the absence of molecular confirmation is presented (case 2). The diagnosis was supported by a highly characteristic T^-^B^+^NK^+^ SCID immunophenotype, a compatible clinical presentation (including disseminated *Candida parapsilosis* infection in early infancy), and the known high prevalence of the CD3D founder variant in Mexican LGM communities. In line with the contemporary Primary Immune Deficiency Treatment Consortium (PIDTC) diagnostic criteria for SCID, molecular confirmation is not strictly mandatory when compatible clinical and immunological findings are present (31). Notably, Case 3 — previously reported by Castaño-Jaramillo et al. as part of a larger Mexican IEI cohort in the context of COVID-19 (8) presented with SARS-CoV-2 infection without developing MIS-C, likely reflecting the profoundly attenuated inflammatory capacity inherent to absent T-cell immunity.

Adenine base editing targeting the LGM CD3D founder variant has achieved 71.2% correction efficiency in human HSPCs and 88% reversion in CD34^+^ cells in murine xenograft models, representing a promising curative gene therapy approach for this population (32).

The genetic heterogeneity of SCID in LGM communities extends beyond ADA and *CD3D* deficiency. Reid et al. recently reported a female LGM infant with hypomorphic RAG1-associated SCID carrying a homozygous *RAG1* c.527G>T variant (her older brother shared the same genotype), who presented at 10 months with cryptosporidium gastroenteritis, CMV viremia refractory to multiple antiviral agents including ganciclovir, valganciclovir, foscarnet, and cidofovir, as well as five maternally derived CMV-specific cytotoxic T lymphocyte infusions. She also had evidence of inflammatory bowel disease on colonoscopy. The patient underwent unconditioned haploidentical peripheral stem cell transplantation with TCRαβ^+^/CD19^+^ depletion at 18 months, achieving rising T cell chimerism from 34% at three months to 80% at one year post-HCT, with CMV clearance and GI disease resolution — a remarkably positive outcome that expands the phenotypic and genetic spectrum of SCID-associated IEI in LGM populations (33).

### 4.2 ZAP-70 deficiency: a well-characterized LGM founder immunodeficiency

ZAP-70 deficiency, caused by the founder splice-site variant c.1624–11G>A, is one of the most extensively characterized LGM IEIs, producing selective CD8^+^ T-cell deficiency with clinically severe combined immunodeficiency despite preserved CD4^+^ T cells (10–12). A global systematic review of 49 patients confirmed this variant as the most frequently identified ZAP70 mutation worldwide — predominantly in Mennonite families — and long-term HCT outcomes in LGM patients showed 100% survival at a median follow-up of 13.5 years with durable immune reconstitution (10, 15).

The clinical consequences of delayed diagnosis were illustrated by Kim et al., who described a Mexican LGM infant requiring emergency HCT for life-threatening infections caused by *Klebsiella pneumoniae*, *Pneumocystis jirovecii*, and parainfluenza virus at four months of age (13). An important limitation of TREC-based NBS for ZAP-70 deficiency was highlighted by Grazioli et al., who documented a seven-month-old LGM infant with rhinovirus-associated acute respiratory distress syndrome and markedly reduced CD8^+^ T cells (20 cells/μL) whose TREC value at birth was normal emphasizing that standard NBS would have failed to identify this patient and that targeted genetic or flow cytometric screening is required (34). To address this gap, Schroeder et al. developed and validated a community-based molecular assay identifying the *ZAP70* c.1624–11G>A genotype in 125 LGM participants: 7 were confirmed affected, 39 were heterozygous carriers, and 79 carried a normal genotype, demonstrating the feasibility and yield of targeted population screening (14). Long-term transplant outcomes were characterized by Cuvelier et al., who reported 100% survival in eight patients who underwent HCT for ZAP-70 deficiency, with a median follow-up of 13.5 years and durable immune reconstitution regardless of graft source or conditioning regimen (15). These data firmly establish HCT as a curative modality for this condition and reinforce the importance of pre-symptomatic diagnosis.

### 4.3 X-linked agammaglobulinemia in the LGM population (case 4)

We report the first case to our knowledge of X-linked agammaglobulinemia (XLA) attributable to a hemizygous BTK variant (c.862C>T; p.Arg288Trp) in an LGM individual, expanding the humoral immunodeficiency spectrum in this population. While classical XLA manifests with recurrent bacterial sinopulmonary infections and markedly reduced serum immunoglobulins in male patients, our patient presented with chronic diarrhea, skin abscesses, and perianal ulcers alongside profound agammaglobulinemia and absent B cells, highlighting the broad clinical spectrum of BTK deficiency. Gastrointestinal manifestations, including chronic diarrhea and perianal disease, have been described in patients with XLA and may reflect the role of humoral immunity in maintaining intestinal homeostasis; XLA-associated enteritis can present with features resembling Crohn’s disease even in the absence of histologically confirmed inflammatory bowel disease (35). This case underscores the importance of considering IEI, including humoral deficiencies, in the differential diagnosis of early-onset or atypical gastrointestinal symptoms particularly in patients from genetically at-risk communities such as LGM, where ascertainment bias may otherwise limit recognition.

### 4.4 G6PC3 deficiency: expanding the LGM phagocyte disorder spectrum (case 1)

In our series the first *G6PC3* deficiency (severe congenital neutropenia type 4) case in a Mexican LGM patient population is described. The recurrence of the identical variant c.482G>A across geographically distant LGM communities in Argentina (23) and Mexico strongly suggests a shared ancestral allele, consistent with the propagation of founder variants through successive LGM migration waves from Canada. *G6PC3* deficiency is a monogenic immunometabolic disorder in which defective glucose-6-phosphatase activity in neutrophils impairs endoplasmic reticulum homeostasis, resulting in accelerated apoptosis and severe neutropenia (36). The syndromic extra-hematologic manifestations — including congenital heart defects, urogenital anomalies, and prominent superficial veins — frequently divert initial clinical attention and contribute to diagnostic delay. G-CSF therapy remains the cornerstone of management, as exemplified by our patient, who has remained clinically stable at eight years of age on continuous G-CSF treatment.

Beyond *G6PC3* deficiency, phagocyte disorders in LGM populations include p14 (LAMTOR2) deficiency, a primary immunodeficiency caused by a homozygous point mutation in the 3’ untranslated region of the *LAMTOR2* gene, first characterized by Bohn et al. in a Mennonite family (37). The clinical phenotype encompassed short stature, hypopigmented skin, coarse facial features, recurrent bronchopulmonary infections caused by *Streptococcus pneumoniae*, and severe congenital neutropenia (ANC <500/μL) with intact bone marrow maturation, responsive to low-dose G-CSF therapy. This condition illustrates that the LGM phagocyte disorder spectrum is not limited to a single gene and warrants broad molecular evaluation in affected individuals.

### 4.5 Atypical phenotypes in DNA repair defects: ATM and bloom syndrome

The ATM founder variant c.6200C>A (p.Ala2067Asp) causes the “A-T Winnipeg” phenotype: early-onset dystonia with late or absent ataxia, typically without telangiectasias or immunodeficiency, creating substantial diagnostic delay that in several LGM patients resulted in diagnosis only after lymphoid malignancy presentation (16–20). Nakamura et al. confirmed functional impairment of DNA double-strand break repair in ten LGM patients homozygous for this variant, emphasizing that ATM testing and systematic cancer surveillance should be pursued in any LGM patient with movement disorders regardless of the classical A-T triad (19).

Bloom syndrome caused by biallelic BLM variants has been documented in LGM individuals, with chromosomal instability studies confirming the diagnosis in Mennonite siblings and an LGM-derived cell line, extending the chromosomal instability and cancer-predisposition burden of this population (21, 22).

### 4.6 Autoinflammatory disease: mevalonate kinase deficiency

MKD in LGM populations is linked to the MVK I268T founder variant (c.803T>C), initially associated with approximately 20% residual kinase activity (38). However, homozygosity for this variant caused severe neonatal disease — including cholestatic liver disease with intrahepatic bile duct paucity requiring HCT at three months — illustrating the incomplete genotype-phenotype correlation and clinical unpredictability of biallelic founder variants in LGM communities (39).

### 4.7 Bone marrow failure syndromes in LGM communities

Fanconi anemia caused by the FANCC c.67delG ancestral Dutch founder allele has been documented in LGM communities in Canada and Mexico, typically presenting with mild skeletal phenotype and bone marrow failure, with identical haplotype origin confirmed across geographically distant communities (40, 41). A telomere biology disorder attributable to a TERT K570N founder variant was characterized in a six-generation LGM kindred, manifesting as aplastic anemia, myelodysplastic syndrome, and macrocytosis across generations; functional studies confirmed haploinsufficiency and telomere shortening, supporting a shared LGM founder effect (42, 43).

### 4.8 Newborn screening and population-targeted diagnostic strategies

The cumulative evidence from LGM-associated IEI provides a compelling rationale for implementing population-specific NBS and genetic screening programs in these communities. NBS for SCID using TREC quantification enables pre-symptomatic identification before the onset of life-threatening infections, allowing timely and optimally conditioned HCT. This program has been successfully implemented in the United States and other countries. Recently, Anchoo et al. reported a significant improvement in overall survival among SCID patients following the introduction of a national NBS program in Israel (44). Implementing a similar initiative in Mexico is critical, especially, as shown here, for high-risk populations such as the LGM population.

A comprehensive approach for LGM communities should therefore integrate: (i) universal TREC-based NBS; (ii) reflexive targeted genotyping for documented LGM founder variants upon abnormal or borderline NBS results; (iii) proactive cascade genetic screening within families of affected individuals; and (iv) clinical awareness programs among primary care providers serving LGM settlements. Implementing such a strategy in Mexico, where approximately 65 LGM communities are established (3, 4), requires coordinated public health investment but is feasible given the relatively small population size and the availability of cost-effective molecular assays.

### 4.9 Differential diagnosis and the broader autosomal recessive disease burden

The diagnostic approach in LGM communities must extend beyond IEI to encompass the full spectrum of autosomal recessive conditions enriched by founder effects. Several metabolic disorders prevalent in LGM populations including maple syrup urine disease, propionic acidemia, mevalonic aciduria, cystinosis, phenylalanine hydroxylase deficiency, and Salla disease (25, 26) can present with clinical features that overlap substantially with IEI, including recurrent infections, failure to thrive, neurodevelopmental delay, and systemic inflammatory manifestations. Awareness of this expanded differential is essential to avoid both under-diagnosis of IEI and misclassification of metabolic conditions as infectious or immunological disease. Population-informed diagnostic panels that incorporate both IEI-related genes and the most prevalent LGM metabolic disease alleles represent the most efficient strategy for genetically isolated communities. Genetic counseling services specifically tailored to LGM cultural and linguistic context are equally indispensable to ensure appropriate uptake of testing and informed reproductive decision-making. .

### 4.10 Limitations

Several limitations of the present study should be acknowledged. First, this is a retrospective case series from two tertiary referral centers; ascertainment bias is therefore unavoidable, and the true prevalence of IEI in Mexican LGM communities cannot be estimated from these data. Furthermore, in the absence of population-based registries that specifically track LGM communities in Mexico, formal incidence, or prevalence estimates of IEI in this population cannot be derived from the present series. Second, molecular confirmation was not obtained for Case 2. The diagnosis of CD3δ deficiency in this patient remains presumptive, based on the T^-^B^+^NK^+^ immunophenotype, clinical presentation, and known population prevalence. This is a critical limitation given the genetic focus of the manuscript, and all conclusions derived from or referencing Case 2 must be interpreted accordingly. Third, the literature review, while comprehensive through August 2025 and including multiple databases and the Amish-Mennonite-Hutterite Genetic Disorders Database, was narrative rather than systematic in synthesis, and may not capture all published cases. Fourth, outcome data for cases identified in the literature was incomplete in several reports, limiting the assessment of long-term prognosis. Finally, the geographic scope of this series — focused on Mexican LGM communities — may not fully represent the IEI burden across all LGM settlements in Canada, the United States, and Latin America. Prospective, multicenter, population-based registries specifically designed for LGM communities would address these limitations and provide the epidemiological data necessary to inform public health policy.

## 5 Conclusion

This case series documents four distinct inborn errors of immunity in Low-German Mennonite patients from Mexico and integrates them with a comprehensive review of previously reported IEI in this population. Taken together, the data reveal a number of pathogenic variants in genes governing T-cell development (*CD3D*, *ZAP70*), B-cell differentiation (*BTK*), phagocyte function (*G6PC3*, *LAMTOR2*), DNA repair (*ATM*, *BLM*, *FANCC*), autoinflammatory regulation (*MVK*), and telomere maintenance (*TERT*) that accumulate within LGM communities through endogamy and consanguinity, generating a high-risk genetic landscape for severe IEI.

Early diagnosis, enabled by targeted newborn screening, cascade family testing, and accessible next-generation sequencing, is critical to improving outcomes. Timely clinical intervention — including hematopoietic cell transplantation, immunoglobulin replacement, and G-CSF therapy — must not be delayed while awaiting definitive molecular diagnosis, as these treatments are lifesaving even in the absence of a confirmed genetic variant. Public health policies should prioritize the implementation of NBS programs and culturally adapted genetic counseling services for LGM communities in Mexico and throughout Latin America. Ongoing research to characterize population-specific pathogenic variants will continue to refine early diagnostic strategies and inform the development of personalized therapeutic approaches, including emerging gene editing technologies targeting LGM founder alleles.

## Data Availability

The raw data supporting the conclusions of this article will be made available by the authors, without undue reservation.
